# Unwinding of Continuous Medicaid Coverage Among Pediatric Community Health Center Patients

**DOI:** 10.1001/jamanetworkopen.2024.58155

**Published:** 2025-02-06

**Authors:** Wyatt P. Bensken, Jenine Dankovchik, Hannah L. Fein, Gabrielle Duhon, Marion R. Sills

**Affiliations:** 1 Department of Research, OCHIN, Portland, Oregon; 2 Department of Population and Quantitative Health Sciences, Case Western Reserve University, Cleveland, Ohio; 3 Department of Pediatrics, University of Colorado, Anschutz Medical Campus, Aurora

## Abstract

**Importance:**

During the COVID-19 pandemic public health emergency, states provided continuous Medicaid coverage to enrollees. In April 2023, states began to unwind this continuous coverage, prompting concern about the impact of this on pediatric patients.

**Objective:**

To examine loss of coverage during the unwinding of continuous Medicaid coverage among pediatric patients seen at community-based health care organizations.

**Design, Setting, and Participants:**

This cohort study with statistical analysis in November 2024 included pediatric patients from a multistate network of community-based health care organizations. Participants were aged up to 17 years at both their last Medicaid-insured visit during the continuous coverage period and at their first visit during the unwinding period (April 1, 2023, to March 31, 2024).

**Exposures:**

Age, sex, race and ethnicity, language, and medical complexity.

**Main Outcome and Measures:**

The main outcome was Medicaid disenrollment to uninsured status during the unwinding period. Logistic regression was used to estimate the odds of ever being uninsured during unwinding, and Cox proportional hazards regression models were used to examine the time to uninsured status from the start of unwinding in each patient’s state of residence through the end of March 2024. Associations between age, sex, race and ethnicity, language, and medical complexity and the outcome were assessed.

**Results:**

Among 450 146 pediatric patients, mean (SD) patient age was 8.11 (5.07) years and 50.1% were male. Overall, 8.7% were disenrolled from Medicaid to uninsured status. Patients aged 12 to 17 years had the highest estimated disenrollment among age groups **(**10.5%), but after adjustment, all other age groups had lower odds of disenrollment compared with those younger than 1 year. Females had higher odds of disenrollment (adjusted odds ratio [AOR], 1.15 [95% CI, 1.13-1.18]; adjusted hazard ratio [AHR], 1.14 [95% CI, 1.12-1.17]) than males. American Indian or Alaska Native patients had higher odds of disenrollment (AOR, 1.95 [95% CI, 1.81-2.09]; AHR, 1.81 [95% CI, 1.05-3.13]) than White patients, with estimated disenrollment of 17.1% vs 9.4%. Compared with patients with low medical complexity, those with either chronic but noncomplex (AOR, 1.83 [95% CI, 1.79-1.88]; AHR, 1.80 [95% CI, 1.44-2.27]) or complex chronic (AOR, 1.95 95% CI, 1.89-2.00]; AHR, 1.92 [95% CI, 1.67-2.21]) medical complexity had higher odds of disenrollment.

**Conclusions and Relevance:**

In this cohort study of previously Medicaid-insured pediatric patients seen at community-based health care organizations, a meaningful proportion of patients were disenrolled to uninsured status during the unwinding of continuous Medicaid coverage, with associated differences by demographic and clinical characteristics. This could impact access to care and health outcomes for the patients.

## Key Points

**Question:**

What was the magnitude of coverage loss during the unwinding of COVID-19 continuous Medicaid coverage among pediatric patients seen at community-based health care organizations?

**Findings:**

In this cohort study of 450 146 pediatric patients, 9% were disenrolled from Medicaid to uninsured status through March 31, 2024. Those who experienced greater disenrollment included patients aged 12 to 17 years, females, American Indian or Alaska Native individuals, and patients with higher medical complexity.

**Meaning:**

The study found that a meaningful proportion of pediatric patients lost coverage during the unwinding of continuous Medicaid coverage, with differences by demographic and clinical characteristics.

## Teaser

This cohort study estimates Medicaid disenrollment to uninsured status among pediatric patients seen at community-based health care organizations during unwinding of continuous Medicaid coverage after the COVID-19 pandemic.

## Introduction

Under the US federal COVID-19 Public Health Emergency and the Families First Coronavirus Response Act, states received additional Medicaid funding and were prohibited from removing patients from Medicaid through eligibility redeterminations.^1,2^ After this 3-year period of continuous Medicaid coverage, there was a mandated end of continuous coverage beginning April 2023. As states resumed eligibility redeterminations in a process termed the *unwinding* of continuous Medicaid coverage,^1,2,3^ stakeholders raised concerns about the detrimental impact this would have on access to care^4,5,6,7^ and outcomes among pediatric patients.^8,9,10,11,12,13^ Despite the more generous eligibility criteria for pediatric patients, which may temper the scale of disenrollments, these concerns were driven by how critical Medicaid is to pediatric health care: 40% of pediatric patients are covered by Medicaid,^14^ although even this may be an underestimate.^15^ As of October 2024, there was a net decrease of 14.7 million Medicaid beneficiaries, one-third of whom (>5.5 million) were youths.^16^

In addition to patient-level effects, unwinding is also expected to negatively impact health centers that serve Medicaid-insured populations, including the 1 in 9 pediatric patients who are cared for by community health centers.^17^ Community-based health care organizations are the frontline primary care organizations serving systemically marginalized patients in the US and help overcome barriers to care, such as cost, distance, and preferred language.^18^ One study reported that 16.7% of adult Medicaid-insured patients seen at community-based health care organizations lost coverage during unwinding, with disparities by age, race and ethnicity, and co-occurring conditions.^19^ To our knowledge, this has yet to be examined among pediatric patients who receive care from community-based health care organizations. In this study, we examined the scope of Medicaid disenrollment to uninsured status during the unwinding of continuous Medicaid coverage among pediatric patients seen at community-based health care organizations.

## Methods

This cohort study was approved by the Advarra institutional review board, with a waiver of informed consent due to the minimal risk. We followed the Strengthening the Reporting of Observational Studies in Epidemiology (STROBE) reporting guideline (eFigure 1 in Supplement 1).

### Data Source and Inclusion Criteria

We used electronic health record (EHR) data from OCHIN, a nonprofit organization with a fully hosted and shared EHR platform for a nationwide network of community-based health care organizations^20^ that is part of the Accelerating Data Value Across a National Community Health Center Network clinical research network.^21^ These organizations serve patients regardless of ability to pay and therefore have a higher proportion of minoritized populations compared with the US population.

Our study focused on pediatric patients aged 0 to 17 years as of their last visit during the period when Medicaid coverage was continuous (January 1, 2021, to March 31, 2023) and their first visit during the unwinding period (April 1, 2023, to March 31, 2024). During the continuous-coverage period, we identified patients who accessed ambulatory or telehealth services and, of that group, those who had visits during both the continuous coverage period and the unwinding period and thus were eligible for this study (eTable in Supplement 1 shows a comparison of these populations). We further required that these visits be at organizations that provided behavioral health or mental health services; community health centers; free clinics; public health departments; rural health centers; specialty clinics; Indian Health Service, tribal, or urban Indian organizations; or social services institutions. Of the identified patients, we limited the study population to those who were Medicaid insured at their last visit during continuous coverage. To further refine the sample, we included only patients from states with substantial representation (>1000 patients) in the OCHIN network: Alabama, Arkansas, California, Connecticut, Georgia, Idaho, Illinois, Indiana, Massachusetts, Minnesota, North Carolina, New Jersey, Ohio, Oklahoma, Oregon, South Carolina, Texas, Washington, and Wisconsin. Since disenrollment began, 3 states (Kentucky, North Carolina, and New Mexico) have approved waivers^22^ to extend pediatric patients’ eligibility and delay redeterminations. However, given the timing of these waivers (redeterminations in these states had already begun) and observed decreases in Medicaid enrollment among youths,^16^ we did not exclude any states but rather statistically accounted for state-level variation.

### Study Outcomes

The outcome for this study was disenrollment from Medicaid, operationalized as being Medicaid insured at the last visit during continuous coverage and then having at least 1 uninsured visit during the unwinding period. At each clinical encounter, a patient’s insurer was recorded and then categorized as Medicaid, Medicare, other public, other or unknown, private, or uninsured. Pediatric patients covered under the Children’s Health Insurance Program (CHIP) were categorized as having Medicaid. These insurer data have been operationalized and used previously in research in the OCHIN network.^23,24,25^

### Independent Variables

The 6 independent variables for this study are as follows: drivers of correct ascertainment of payer status (number of visits), drivers of utilization patterns and Medicaid eligibility (age and medical complexity), and factors that represent populations with health disparity (sex, race and ethnicity, and language).

First, we calculated the number of visits a patient had during the unwinding period. Given that our data were based on health care encounters, our ability to determine insurance status was directly linked to receiving care. We only counted encounters that occurred before becoming uninsured to avoid introducing bias by conditioning on a covariate that occurred after the outcome. This first independent variable was used as a covariate across models. Second, age was calculated as age at the patient’s last visit during continuous coverage (on or before March 31, 2023). We included age as a covariate because it is related to the number of recommended visits as well as to state-level Medicaid eligibility requirements, with the youngest age group (<1 year) set as the reference group, as this group often has the most generous eligibility criteria.

Third, sex was documented as sex assigned at birth; possible categories were ambiguous, female, male, no information, and unknown. Due to small cell size counts, we collapsed these categories to female, male, and other or unknown. Fourth, a combined race and ethnicity variable was created from 2 separate variables documenting a patient’s race and ethnicity. Categories for race included American Indian or Alaska Native, Asian, Black or African American, Native Hawaiian or Other Pacific Islander, White, multiracial, and unknown or other (included patients who declined to answer, for whom there was no information, or who reported other as their race). Patients who reported being Hispanic were classified as Hispanic, while all others were classified as their reported race. An exception to this was for those who identified as American Indian or Alaska Native, who were classified as their race regardless of ethnicity given that American Indian or Alaska Native is the racial group most likely to be racially misclassified^26,27^ and most likely to identify as multiracial.^28^ Expanding the definition of the American Indian or Alaska Native population to include Hispanic individuals, while not eliminating the challenge of racial misclassification, offered a strategy to mitigate its impact. Fifth, we reported the patient’s preferred language, categorized as English, Spanish, or other. Sixth, we included a measure of medical complexity to account for the chronic disease burden that may affect Medicaid eligibility and enrollment directly (via eligibility) or indirectly (via greater contact with the health care system and social support to assist with enrollment). We operationalized medical complexity using the Pediatric Medical Complexity Algorithm, which uses diagnosis codes to categorize patient medical complexity as nonchronic, chronic but noncomplex, or complex chronic.^29^

### Statistical Analysis

We used 2 complementary analytic approaches to assess the association between sociodemographic factors and Medicaid disenrollment during the unwinding period. First, we used a generalized linear model with a logit link function to estimate the odds of ever being uninsured during the unwinding period. Second, we used Cox proportional hazards regression models to account for right-censoring. The time-to-event analysis period began on April 1, 2023, the earliest date when disenrollment to uninsured status could take place, and continued until either the first recorded encounter at which a patient was uninsured (event = 1) or the last documented encounter within the unwinding period (event = 0, censored). The first approach allowed assessment of whether specific subgroups of patients had higher odds of losing coverage, while the second approach considered differences in time to disenrollment and accounted for differential follow-up time and timing of visits among patients.

For both approaches, we examined the associations between age, sex, race and ethnicity, language, and medical complexity and the outcome. All models adjusted for the number of visits, and the models for age, sex, race and ethnicity, and language further adjusted for medical complexity. We chose a series of only partially adjusted models to avoid overadjusting our analysis. These adjustments were made because observing coverage changes is dependent on visits and pediatric Medicaid coverage is influenced by medical complexity. All models included a random intercept for state or accounted for state-level clustering. We used a 2-sided significance level (α) of 0.05, and we present adjusted odds ratio (AOR) and adjusted hazard ratio (AHR) estimates and their corresponding 95% CIs. We also calculated^30^ the estimated disenrollment percentage for the primary variables. Data were analyzed in November 2024 using R, version 4.4.2 (R Project for Statistical Computing).

## Results

Of 970 711 pediatric patients who accessed ambulatory or telehealth services during the continuous-coverage period, 536 769 (55.3%) had visits during both the continuous-coverage period and the unwinding period. A total of 535 107 unique patients were seen at 182 organizations that met the inclusion criteria; 477 531 of these patients were Medicaid insured at their last visit during continuous coverage. Including patients only from states with more than 1000 patients in the OCHIN network yielded a final analytic cohort of 450 146 pediatric patients who were Medicaid insured during the period of continuous coverage and also had a visit during the unwinding period. Mean (SD) patient age was 8.11 (5.07) years. One-third of the patients (151 479 [33.7%]) were 6 to 11 years of age, 31 008 (6.9%) were younger than 1 year, 224 747 (49.9%) were female, and 225 354 (50.1%) were male. A total of 6048 (1.3%) were American Indian or Alaska Native; 22 066 (4.9%), Asian; 62 060 (13.8%), Black or African American; 234 901 (52.2%), Hispanic; 391 (0.1%), Native Hawaiian or Other Pacific Islander; 79 223 (17.6%), White; 5381 (1.2%), multiracial; and 40 076 (8.9%), other or unknown race and ethnicity (Table 1). Over half of patients (241 555 [53.7%]) preferred care in English; 176 530 (39.2%) preferred care in Spanish (Table 1). A total of 174 494 patients (38.8%) had a medical complexity status classified as nonchronic; 126 344 (28.1%), as chronic but noncomplex; and 149 308 (33.2%), as complex chronic (Table 1).

**Table 1.** Description of the Study Population Stratified by Disenrollment From Medicaid to Uninsured Status During Unwinding

Characteristic	Patients, No. (%)		
	Study population (N = 450 146)	Not disenrolled (n = 410 852 [91.3%])	Disenrolled (n = 39 294 [8.7%])
Age, y			
<1	31 008 (6.9)	27 884 (6.8)	3124 (8.0)
1-5	127 857 (28.4)	118 593 (28.9)	9264 (23.6)
6-11	151 479 (33.7)	139 247 (33.9)	12 232 (31.1)
12-17	139 802 (31.1)	125 128 (30.5)	14 674 (37.3)
Sex			
Female	224 747 (49.9)	204 285 (49.7)	20 462 (52.1)
Male	225 354 (50.1)	206 532 (50.3)	18 822 (47.9)
Other or unknown	43 (<0.1)	NR^a^	NR^a^
Race and ethnicity			
American Indian or Alaska Native	6048 (1.3)	5014 (1.2)	1034 (2.6)
Asian	22 066 (4.9)	20 775 (5.1)	1291 (3.3)
Black or African American	62 060 (13.8)	56 471 (13.7)	5589 (14.2)
Hispanic^b^	234 901 (52.2)	215 201 (52.4)	19 700 (50.1)
Native Hawaiian or Other Pacific Islander	391 (0.1)	358 (0.1)	33 (0.1)
White	79 223 (17.6)	71 756 (17.5)	7467 (19.0)
Multiracial	5381 (1.2)	4843 (1.2)	538 (1.4)
Other or unknown^c^	40 076 (8.9)	36 434 (8.9)	3642 (9.3)
Preferred language			
English	241 555 (53.7)	220 090 (53.6)	21 465 (54.6)
Spanish	176 530 (39.2)	160 896 (39.2)	15 634 (39.8)
Other	32 061 (7.1)	29 866 (7.3)	2195 (5.6)
Medical complexity			
Nonchronic	174 494 (38.8)	163 587 (39.8)	10 907 (27.8)
Chronic, noncomplex	126 344 (28.1)	113 449 (27.6)	12 895 (32.8)
Chronic, complex	149 308 (33.2)	133 816 (32.6)	15 492 (39.4)
Visits during unwinding before becoming uninsured, No.			
1	152 601 (33.9)	132 226 (32.2)	20 375 (51.9)
2	95 847 (21.3)	90 083 (21.9)	5764 (14.7)
3	64 317 (14.3)	60 520 (14.7)	3797 (9.7)
4	43 404 (9.6)	40 824 (9.9)	2580 (6.6)
≥5	93 977 (20.9)	87 199 (21.2)	6778 (17.2)

Abbreviation: NR, not reported.

^a^ Masked for confidentiality due to small sample sizes.

^b^ Patients who reported being Hispanic were classified as Hispanic; all others were classified as their self-reported race with the exception of American Indian or Alaska Native individuals, who were classified as their race regardless of ethnicity.

^c^ Includes patients who declined to answer, for whom there was no information, or who reported other as their race; exact responses and values are not known.

Of the included pediatric patients, 39 294 (8.7%) were disenrolled from Medicaid to uninsured status during the unwinding period. Unadjusted descriptive statistics showed that disenrolled patients tended to be older, to prefer care in English, and to have chronic complex medical complexity status (Table 1). After adjusting for number of visits, medical complexity, and state-level clustering, differences persisted by age, sex, race and ethnicity, language, and medical complexity. We found that patients aged 12 to 17 years had the highest estimated disenrollment percentage at 10.5%. After adjustment, compared with those younger than 1 year, all other age groups had lower odds of being disenrolled to uninsured status (eg, 12-17 years: AOR, 0.71; 95% CI, 0.68-0.74). Female patients had higher odds (AOR, 1.15; 95% CI, 1.13-1.18), a higher AHR (1.14; 95% CI, 1.12-1.17), and higher estimated disenrollment (9.1% vs 8.4%) compared with male patients (Table 2 and Table 3). Unadjusted Kaplan-Meier curves are presented in eFigures 2 to 7 in Supplement 1.

**Table 2.** AORs and Estimated Disenrollment From Medicaid to Uninsured Status^a^

Characteristic	AOR (95% CI)	Estimated disenrollment, % (95% CI)
Age, y^b^		
<1	1 [Reference]	10.1 (8.6-11.5)
1-5	0.52 (0.50-0.55)	7.2 (6.2-8.3)
6-11	0.56 (0.53-0.58)	8.1 (6.9-9.3)
12-17	0.71 (0.68-0.74)	10.5 (9.0-12.0)
Sex^b^		
Female	1.15 (1.13-1.18)	9.1 (7.8-10.4)
Male	1 [Reference]	8.4 (7.2-9.5)
Other or unknown	2.71 (1.72-4.27)	22.2 (14.3-30.2)
Race and ethnicity^b^		
American Indian or Alaska Native	1.95 (1.81-2.09)	17.1 (14.8-19.4)
Asian	0.59 (0.56-0.63)	5.9 (5.0-6.7)
Black or African American	0.78 (0.75-0.81)	9.0 (7.8-10.3)
Hispanic	0.93 (0.90-0.96)	8.4 (7.2-9.6)
Native Hawaiian or Other Pacific Islander	0.80 (0.62-1.03)	8.4 (6.3-10.6)
White	1 [Reference]	9.4 (8.1-10.7)
Multiracial	1.07 (0.97-1.17)	10.0 (8.5-11.5)
Other or unknown^c^	0.99 (0.95-1.03)	9.1 (7.8-10.4)
Preferred language^b^		
English	1 [Reference]	8.9 (7.7-10.1)
Spanish	1.12 (1.09-1.14)	8.9 (7.6-10.1)
Other	0.74 (0.70-0.77)	6.8 (5.8-7.9)
Medical complexity^d^		
Nonchronic	1 [Reference]	6.3 (5.4-7.1)
Chronic, noncomplex	1.83 (1.79-1.88)	10.2 (8.9-11.5)
Chronic, complex chronic	1.95 (1.89-2.00)	10.4 (9.1-11.7)

Abbreviation: AOR, adjusted odds ratio.

^a^ The table shows results for 5 different logistic regression models: 1 for each independent variable of interest, and all accounting for clustering within states.

^b^ Adjusted for medical complexity and the number of visits.

^c^ Includes patients who declined to answer, for whom there was no information, or who reported other as their race.

^d^ Adjusted for the number of visits.

**Table 3.** AHRs and Estimated Disenrollment From Medicaid to Uninsured Status^a^

Characteristic	AHR (95% CI)	Estimated disenrollment, % (95% CI)
Age, y^b^		
<1	1 [Reference]	10.1 (8.0-12.2)
1-5	0.48 (0.42-0.55)	7.3 (5.4-9.1)
6-11	0.50 (0.40-0.62)	8.1 (5.3-10.9)
12-17	0.68 (0.52-0.90)	10.5 (6.3-14.8)
Sex^b^^,^^c^		
Female	1.14 (1.12-1.17)	9.1 (7.6-10.6)
Male	1 [Reference]	8.4 (7.1-9.7)
Race and ethnicity^b^		
American Indian or Alaska Native	1.81 (1.05-3.13)	17.1 (7.8-26.5)
Asian	0.65 (0.50-0.86)	5.9 (4.7-7.0)
Black or African American	0.89 (0.67-1.18)	9.0 (7.0-11.0)
Hispanic	0.90 (0.69-1.17)	8.4 (6.8-10.0)
Native Hawaiian or Other Pacific Islander	1.35 (0.87-2.09)	8.5 (5.2-11.7)
White	1 [Reference]	9.4 (8.0-10.9)
Multiracial	1.05 (0.88-1.25)	10.0 (8.2-11.8)
Other or unknown^d^	1.02 (0.73-1.44)	9.1 (6.9-11.3)
Language^b^		
English	1 [Reference]	8.9 (7.5-10.3)
Spanish	1.02 (0.85-1.22)	8.9 (7.7-10.0)
Other	0.83 (0.67-1.01)	6.9 (5.1-8.6)
Medical complexity^e^		
Nonchronic	1 [Reference]	6.3 (6.1-6.4)
Chronic, noncomplex	1.80 (1.44-2.27)	10.2 (7.6-12.8)
Chronic, complex chronic	1.92 (1.67-2.21)	10.4 (8.5-12.2)

Abbreviation: AHR, adjusted hazard ratio.

^a^ The table represents 5 different Cox proportional hazards regression models, 1 for each independent variable of interest, and all accounting for clustering within states.

^b^ Adjusted for medical complexity and the number of visits.

^c^ Other or unknown sex was removed due to model issues.

^d^ Includes patients who declined to answer, for whom there was no information, or who reported other as their race.

^e^ Adjusted for the number of visits.

In our adjusted analyses, we found that patients identifying as American Indian or Alaska Native had higher disenrollment (AOR, 1.95 [95% CI, 1.81-2.09]; AHR, 1.81 [95% CI, 1.05-3.13]) compared with White patients, with estimated disenrollment of 17.1% vs 9.4% (Table 2 and Table 3). Patients who identified as Black or African American, Hispanic, multiracial, or other or unknown race and ethnicity had no consistent differences in estimated disenrollment across models compared with White patients (Table 2 and Table 3). Patients who identified as Asian had lower odds and lower likelihood of disenrollment than White patients, with estimated disenrollment of 5.9%. While 1 approach showed higher odds of disenrollment among Spanish-preferring patients compared with English-preferring patients (AOR, 1.12; 95% CI, 1.09-1.14), our time-to-event model and estimated disenrollment percentages suggested no differences between English- and Spanish-preferring patients.

Compared with patients with medical complexity classified as nonchronic, those with medical complexity classified as either chronic but noncomplex (AOR, 1.83 [95% CI, 1.79-1.88]; AHR, 1.80 [95% CI, 1.44-2.27]) or complex chronic (AOR, 1.95; 95% CI, 1.89-2.00]; AHR, 1.92 [95% CI, 1.67-2.21]) had higher odds and a higher likelihood of disenrollment as well as higher estimated disenrollment of 10.2% and 10.4% respectively, compared with 6.3% for those with nonchronic medical complexity (Table 2 and Table 3).

## Discussion

This cohort study found that in a cohort of 450 146 previously Medicaid-insured pediatric patients who received care at community-based health care organizations, 8.7% were disenrolled from Medicaid to uninsured status during the unwinding period. These findings are important given that these organizations often assist patients in maintaining coverage^31^ and experience narrow financial margins,^32^ meaning that these shifts in patient-level insurance coverage are likely to have a profound impact on ability to deliver care. Specific pediatric patient groups with higher odds of disenrollment to uninsured status included those younger than 1 year, those aged 12 to 17 years, females, American Indian or Alaska Native patients, and those with higher medical complexity.

National data report that during the same period as this study, over 5.5 million pediatric Medicaid patients were disenrolled, and by September 2023, there was a 5.5% decrease in child Medicaid enrollment.^16,33^ While this national estimate includes patients who transitioned from Medicaid to private insurance, our study more narrowly focused on patients who were disenrolled to uninsured status. Given our study’s longer time frame (through March 2024) and that some uninsured patients do not seek care, it is possible that the estimates provided by our research are a lower bound of disenrollment among minoritized populations. Meanwhile, compared with a study of adult patients in community health centers, we found that pediatric patients had lower disenrollment (8.7%) than adult patients (16.7%).^19^ This may be driven by differences in eligibility for Medicaid coverage for adults compared with children, increased attention to maintaining coverage among pediatric patients, and ongoing initiatives to reduce the number of redeterminations and extend coverage for children insured through Medicaid.^34^ Our results are also consistent with recent work that used surveys to assess the scale of unwinding.^35^

The findings from this study point to several specific consequences for pediatric patients who lost coverage during Medicaid unwinding. First, the age and sex findings indicate that adolescent females may experience the greatest consequences from loss of access to preventive care and that this is occurring at a particularly important time in their development.^36^ Second, our findings highlight that American Indian or Alaska Native patients had greater disenrollment than their White counterparts, which is likely to amplify existing inequities in access to health care and outcomes.^37,38,39,40,41,42,43^ Third, we found no consistent difference in disenrollment across preferred languages. This may be reflective of each individual state’s intentional outreach during redeterminations^44^ and the role of these organizations in providing culturally competent and linguistically concordant services.^17,45^ However, our approaches were not uniform in this finding. While logistic regression suggested that Spanish-preferring patients had higher disenrollment, this was not seen in the time-to-event model or the estimated disenrollment percentages. These findings underscore that our logistic regression, while providing valuable estimates over our study period, may be sensitive to right-censoring and selection bias in these data.

We found that pediatric patients with higher levels of medical complexity had greater disenrollment to uninsured status even after adjusting for the number of visits. This partially may have occurred because these patients and their parents or guardians are more likely to seek care and therefore have their insurance status observed, are more likely to make or keep a medical appointment even when uninsured, or are more likely to seek care without knowing their insurance status. Despite our adjustment, we were not able to fully understand these nuanced and complex relationships. Our findings nonetheless underscore that among pediatric patients who are being seen for care, those who have experienced loss of coverage may also be those who are most likely to need consistent and potentially specialty medical care. For this population, the health consequences of Medicaid unwinding may be even more substantial than initially suspected.^13^

We also found that despite more generous eligibility for pediatric patients younger than 1 year, children and adolescents had lower disenrollment than newborns and infants. While our models adjusted for the number of visits prior to becoming uninsured, given that our ability to measure insurance status was dependent on a visit, it is likely that there was residual confounding driven by this approach. Simply, newborns and infants require more preventive visits^46^ and have greater health care utilization,^47^ meaning they are more likely to have a clinical encounter in a given year and thus have greater opportunity to have observed uninsured status. A full exploration of this is provided in the eMethods in Supplement 1.

### Limitations

This study has limitations. The use of encounter-based data in this study precluded the ability to understand the reason for disenrollment (ie, whether patients lost coverage due to administrative or eligibility reasons). Using these encounter-based data also precluded the study of patients who did not return for care. However, given the focus on pediatric patients and the long study time frame, we found that 55.3% of all pediatric patients returned for care during our study period, and those who returned were demographically similar to those who did not (eTable in Supplement 1). Nonetheless, this may remain a lower bound of disenrollment to uninsured status. Furthermore, while our analyses statistically adjusted for state-level variation, we were unable to fully capture the nuanced and complex processes within each state that led to disenrollment. Additionally, we focused our study on the worst potential outcome: disenrollment to uninsured status. We did not examine pediatric patients who transitioned from Medicaid to a private plan or other public coverage. The most substantial limitation of this study was that only patients seen at community-based health care organizations within the OCHIN network were included. While OCHIN members and their patients broadly resemble community health centers and patients nationwide,^17^ this study did not capture all patients or all states and represents a potentially biased sample; however, to our knowledge, no nationwide dataset of this nature exists. Furthermore, this study sample is not reflective of all Medicaid enrollees but rather has a high proportion of the most minoritized populations (eg, 52.2% of the patients in this study were Hispanic vs 32% of all Medicaid-enrolled pediatric patients^48^). When these results are contextualized as being captured in a setting where clinics rely on Medicaid for their funding and thus have an incentive to help patients stay enrolled and often assist in navigating Medicaid,^49^ it is likely that these findings are an underestimation of broader disenrollment to uninsured status.

## Conclusions

In this cohort study of systemically underserved pediatric patients who received care from frontline primary care health care organizations, we identified that during the unwinding of continuous coverage provisions, many pediatric patients were disenrolled from Medicaid to uninsured status. Future work should continue to examine the impact of this disenrollment to uninsured status on patients’ access to care and health outcomes and clinic operations.

## Notes

Supplement 1. **eFigure 1.** STROBE Diagram of Study Inclusion Criteria **eTable.** Description of Study Population, Stratified by Meeting or Not Meeting Inclusion Criteria of Having a Visit During Continuous Coverage and During Unwinding **eMethods.** Additional Analyses Related to Age, Number of Visits, and Disenrollment **eFigure 2.** Kaplan-Meier Curve Demonstrating the Unadjusted Time to Loss of Coverage Among Medicaid-Insured Pediatric Patients **eFigure 3.** Kaplan-Meier Curve Showing the Unadjusted Time to Loss of Coverage Among Medicaid-Insured Pediatric Patients, Stratified by Age **eFigure 4.** Kaplan-Meier Curve Showing the Unadjusted Time to Loss of Coverage Among Medicaid-Insured Pediatric Patients, Stratified by Sex **eFigure 5.** Kaplan-Meier Curve Showing the Unadjusted Time to Loss of Coverage Among Medicaid-Insured Pediatric Patients, Stratified by Race and Ethnicity **eFigure 6.** Kaplan-Meier Curve Showing the Unadjusted Time to Loss of Coverage Among Medicaid-Insured Pediatric Patients, Stratified by Language **eFigure 7.** Kaplan-Meier Curve Showing the Unadjusted Time to Loss of Coverage Among Medicaid-Insured Pediatric Patients, Stratified by Medical Complexity

Supplement 2.
Data Sharing Statement
