# Supplementary material for: Unwinding of Continuous Medicaid Coverage Among Pediatric Community Health Center Patients
Source: JAMA Netw Open. 2025 Feb 6;8(2):e2458155. doi: 10.1001/jamanetworkopen.2024.58155 (PMC11803474; doi:10.1001/jamanetworkopen.2024.58155)
Supplement: Supplement 1. — eFigure 1. STROBE Diagram of Study Inclusion Criteria eTable. Description of Study Population, Stratified by Meeting or Not Meeting Inclusion Criteria of Having a Visit During Continuous Coverage and During Unwinding eMethods. Additional Analyses Related to Age, Number of Visits, and Disenrollment eFigure 2. Kaplan-Meier Curve Demonstrating the Unadjusted Time to Loss of Coverage Among Medicaid-Insured Pediatric Patients eFigure 3. Kaplan-Meier Curve Showing the Unadjusted Time to Loss of Coverage Among Medicaid-Insured Pediatric Patients, Stratified by Age eFigure 4. Kaplan-Meier Curve Showing the Unadjusted Time to Loss of Coverage Among Medicaid-Insured Pediatric Patients, Stratified by Sex eFigure 5. Kaplan-Meier Curve Showing the Unadjusted Time to Loss of Coverage Among Medicaid-Insured Pediatric Patients, Stratified by Race and Ethnicity eFigure 6. Kaplan-Meier Curve Showing the Unadjusted Time to Loss of Coverage Among Medicaid-Insured Pediatric Patients, Stratified by Language eFigure 7. Kaplan-Meier Curve Showing the Unadjusted Time to Loss of Coverage Among Medicaid-Insured Pediatric Patients, Stratified by Medical Complexity [file jamanetwopen-e2458155-s001.pdf]

Supplementary Online Content

Bensken WP, Dankovchik J, Fein HL, Duhon G, Sills MR. Unwinding of continuous Medicaid coverage among pediatric community health center patients. *JAMA Netw Open*. 2025;8(2):e2458155. doi:10.1001/jamanetworkopen.2024.58155

eFigure 1. STROBE Diagram of Study Inclusion Criteria
eTable. Description of Study Population, Stratified by Meeting or Not Meeting Inclusion Criteria of Having a Visit During Continuous Coverage and During Unwinding
eMethods. Additional Analyses Related to Age, Number of Visits, and Disenrollment
eFigure 2. Kaplan-Meier Curve Demonstrating the Unadjusted Time to Loss of Coverage Among Medicaid-Insured Pediatric Patients
eFigure 3. Kaplan-Meier Curve Showing the Unadjusted Time to Loss of Coverage Among Medicaid-Insured Pediatric Patients, Stratified by Age
eFigure 4. Kaplan-Meier Curve Showing the Unadjusted Time to Loss of Coverage Among Medicaid-Insured Pediatric Patients, Stratified by Sex
eFigure 5. Kaplan-Meier Curve Showing the Unadjusted Time to Loss of Coverage Among Medicaid-Insured Pediatric Patients, Stratified by Race and Ethnicity
eFigure 6. Kaplan-Meier Curve Showing the Unadjusted Time to Loss of Coverage Among Medicaid-Insured Pediatric Patients, Stratified by Language
eFigure 7. Kaplan-Meier Curve Showing the Unadjusted Time to Loss of Coverage Among Medicaid-Insured Pediatric Patients, Stratified by Medical Complexity

This supplementary material has been provided by the authors to give readers additional information about their work.

© 2025 Bensken WP et al. *JAMA Network Open*.

eFigure 1. STROBE diagram of study inclusion criteria

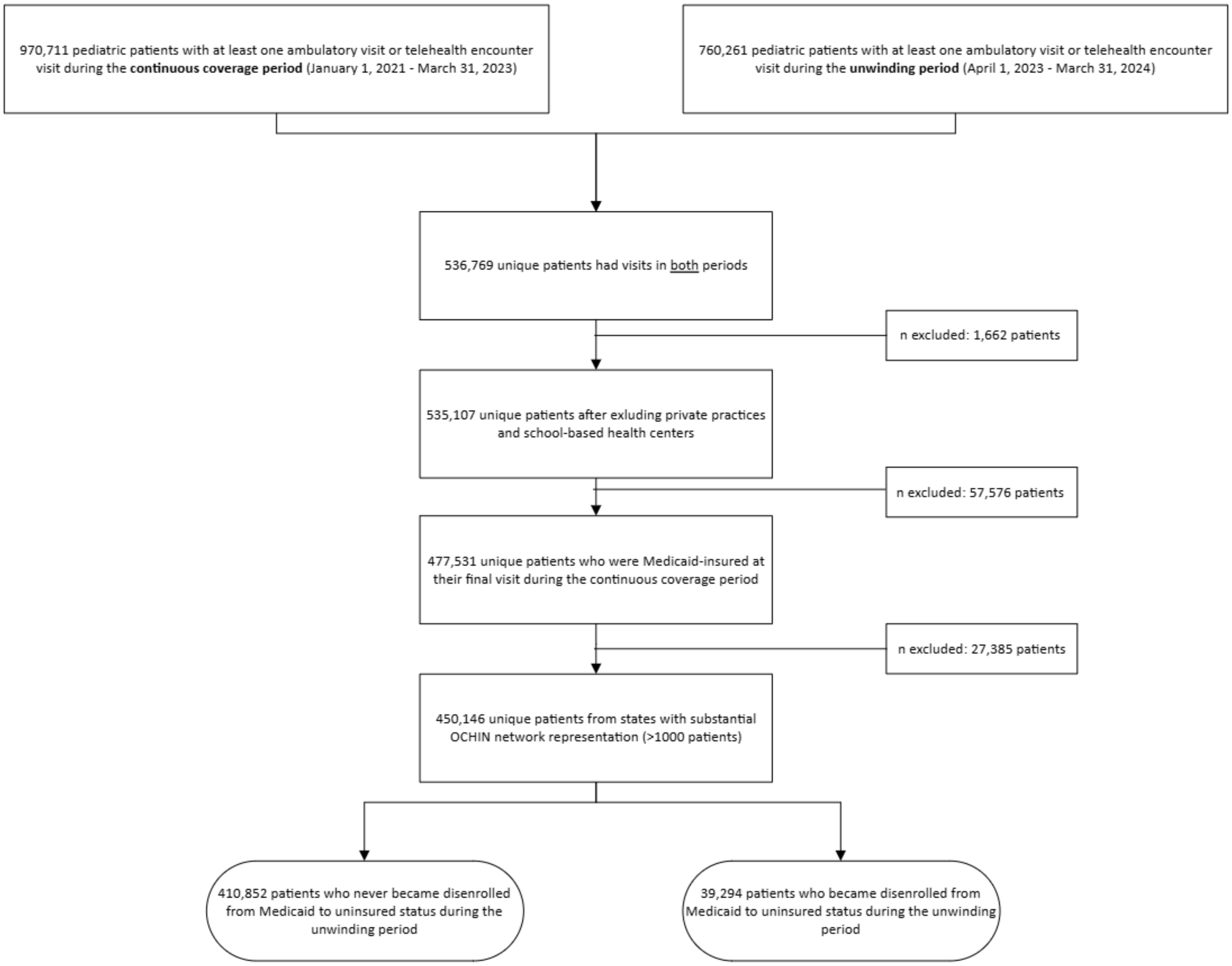

© 2025 Bensken WP et al. *JAMA Network Open*.

eTable. Description of study population, stratified by meeting or not meeting inclusion criteria of having a visit during continuous coverage *and* during unwinding.

n (column %)	**Study Population** n = 450,146	**1 Visit During Continuous Coverage Window** n = 970,711	**Additional Visit During Unwinding** n = 536,769 (55.3%)
**Age**			
< 1	31008 (6.9)	58821 (6.1)	35132 (6.5)
1 to 5	127857 (28.4)	228195 (23.5)	146966 (27.4)
6 to 11	151479 (33.7)	298245 (30.7)	180388 (33.6)
12 to 17	139802 (31.1)	385450 (39.7)	174283 (32.5)
**Sex**			
Female	224747 (49.9)	487619 (50.2)	269378 (50.2)
Male	225354 (50.1)	482593 (49.7)	267263 (49.8)
Other/Unknown	43 (0.0)	499 (0.1)	128 (0.0)
**Race and Ethnicity**			
American Indian or Alaskan Native	6048 (1.3)	12474 (1.3)	7815 (1.5)
Asian	22066 (4.9)	50296 (5.2)	25575 (4.8)
Black or African American	62060 (13.8)	146099 (15.1)	72254 (13.5)
Hispanic	234901 (52.2)	437151 (45.0)	266366 (49.6)
Multiple race	5381 (1.2)	12844 (1.3)	6936 (1.3)
Native Hawaiian or Other Pacific Islander	391 (0.1)	2536 (0.3)	458 (0.1)
Other[1]/Unknown	40076 (8.9)	101173 (10.4)	48811 (9.1)
White	79223 (17.6)	208138 (21.4)	108554 (20.2)
**Preferred Language**			
English	241555 (53.7)	603856 (62.2)	303396 (56.5)
Spanish	176530 (39.2)	296531 (30.5)	196916 (36.7)
Other	32061 (7.1)	70324 (7.2)	36457 (6.8)
**Medical Complexity[1]**			
Non-Chronic	174494 (38.8)	461097 (47.5)	209986 (39.1)
Chronic, Non-Complex	126344 (28.1)	253761 (26.1)	152895 (28.5)
Chronic, Complex	149308 (33.2)	255853 (26.4)	173888 (32.4)

[1] The observed lower medical complexity was expected given that these patients have been seen less frequently and less recently contributing less data related to chronic conditions. While it is also likely that patients of lower complexity are seen less frequently, given this study's focus on pediatric patients it is likely that primary care visits are not driven solely by complexity.

© 2025 Bensken WP et al. *JAMA Network Open*.

eMethods. Additional Analyses Related to Age, Number of Visits, and Disenrollment.

Our main analysis revealed a potentially paradoxical relationship between age and disenrollment. To explore this, we conducted several supplementary analyses. First, (1) we examined the association between age and disenrollment when only including a random intercept for state. Second (2) we examined the association between number of visits and disenrollment when only including a random intercept for state. Third, (3) we examined the relationship between age and number of visits without including a random intercept. Fourth, and finally, (4) we estimated the relationship between age and disenrollment while adjusting for the number of visits and including a random intercept for state.

These analyses revealed that younger age was associated with more visits (3), when adjusting for the number of visits all ages had higher odds of disenrollment than <1 year-olds (4). Importantly, we observed a bias *away* from the null between our unadjusted age-disenrollment relationship (1 versus 4) while the relationship between number of visits and disenrollment (2 versus 4) remained the same. Ultimately, this suggests that while the number of visits mediates the relationship between age and disenrollment, this mediation is only partial and there does indeed remain a relationship between age and disenrollment.

1. Unadjusted Association Between Age and Disenrollment

Generalized linear model with a logit link function, including a random intercept for state.

**Age**	**Disenrolled to Uninsured, % (n)**	**Odds Ratio (95% CI)**
< 1	10.1% (3,124)	*Ref*
1 to 5	7.2% (9,264)	0.70 (0.67, 0.73)
6 to 11	8.1% (12,232)	0.78 (0.75, 0.81)
12 to 17	10.5% (14,674)	1.04 (1.00, 1.08)
Total	*8.7% (39,294)*	

2. Unadjusted Association Between Number of Visits and Disenrollment

Generalized linear model with a logit link function, including a random intercept for state.

**Visit Count**	**Disenrolled to Uninsured, % (n)**	**Odds Ratio (95% CI)**
1	13.4% (20,375)	*Ref*
2	6.0% (5,764)	0.42 (0.41, 0.44)
3	5.9% (3,797)	0.42 (0.41, 0.44)
4	5.9% (2,580)	0.42 (0.41, 0.44)
5+	7.2% (6,778)	0.52 (0.51, 0.54)
Total	*8.7% (39,294)*	

3. Unadjusted Association Between Age and Number of Visits

Multinomial log-linear model with a logit link function, including a random intercept for state.

**Percent Disenrolled**					
Age (row %)	*Visit Count*				
	1	2	3	4	5+
< 1	15.3%	12.3%	13.0%	14.4%	45.0%
1 to 5	32.4%	22.5%	15.5%	10.1%	19.4%
6 to 11	36.4%	22.1%	14.1%	9.0%	18.4%

© 2025 Bensken WP et al. *JAMA Network Open*.

12 to 17	36.7%	21.3%	13.7%	8.8%	19.6%
Total	33.9%	21.3%	14.3%	9.6%	20.9%

**Estimate (95% CI)**					
Age	*Visit Count (Outcome)*				
	1	2	3	4	5+
< 1	*Ref*	*Ref*	*Ref*	*Ref*	*Ref*
1 to 5	*Ref*	0.86 (0.82, 0.90)	0.56 (0.54, 0.59)	0.33 (0.32, 0.35)	0.20 (0.20, 0.21)
6 to 11	*Ref*	0.75 (0.72, 0.79)	0.46 (0.44, 0.48)	0.26 (0.25, 0.28)	0.17 (0.17, 0.18)
12 to 17	*Ref*	0.72 (0.69, 0.75)	0.44 (0.42, 0.46)	0.26 (0.24, 0.27)	0.18 (0.18, 0.19)

4. Adjusted Association Between Age and Disenrollment

Generalized linear model with a logit link function, including number of visits and a random intercept for state.

**Term**	**Odds Ratio (95% CI)**
**Age**	
< 1	*Ref*
1 to 5	0.61 (0.58, 0.63)
6 to 11	0.66 (0.63, 0.69)
12 to 17	0.87 (0.84, 0.91)
**Visit Count**	
1	*Ref*
2	0.43 (0.41, 0.44)
3	0.42 (0.40, 0.43)
4	0.42 (0.40, 0.43)
5+	0.50 (0.48, 0.51)

© 2025 Bensken WP et al. *JAMA Network Open*.

eFigure 2. Kaplan-Meier curve demonstrating the unadjusted time-to-loss of coverage among Medicaid-insured pediatric patients.

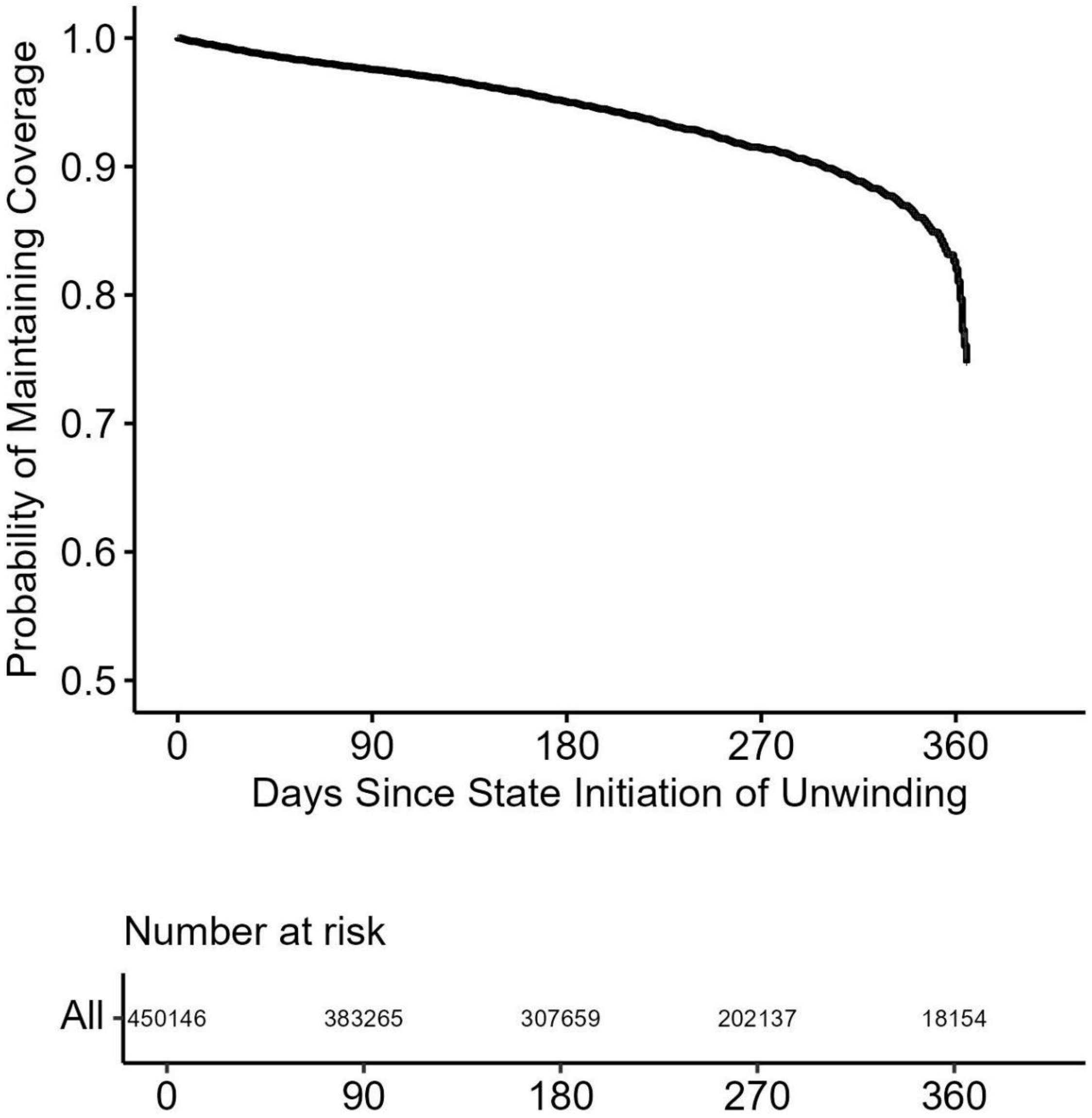

© 2025 Bensken WP et al. *JAMA Network Open*.

eFigure 3. Kaplan-Meier curve showing the unadjusted time-to-loss of coverage among Medicaid-insured pediatric patients, stratified by age.

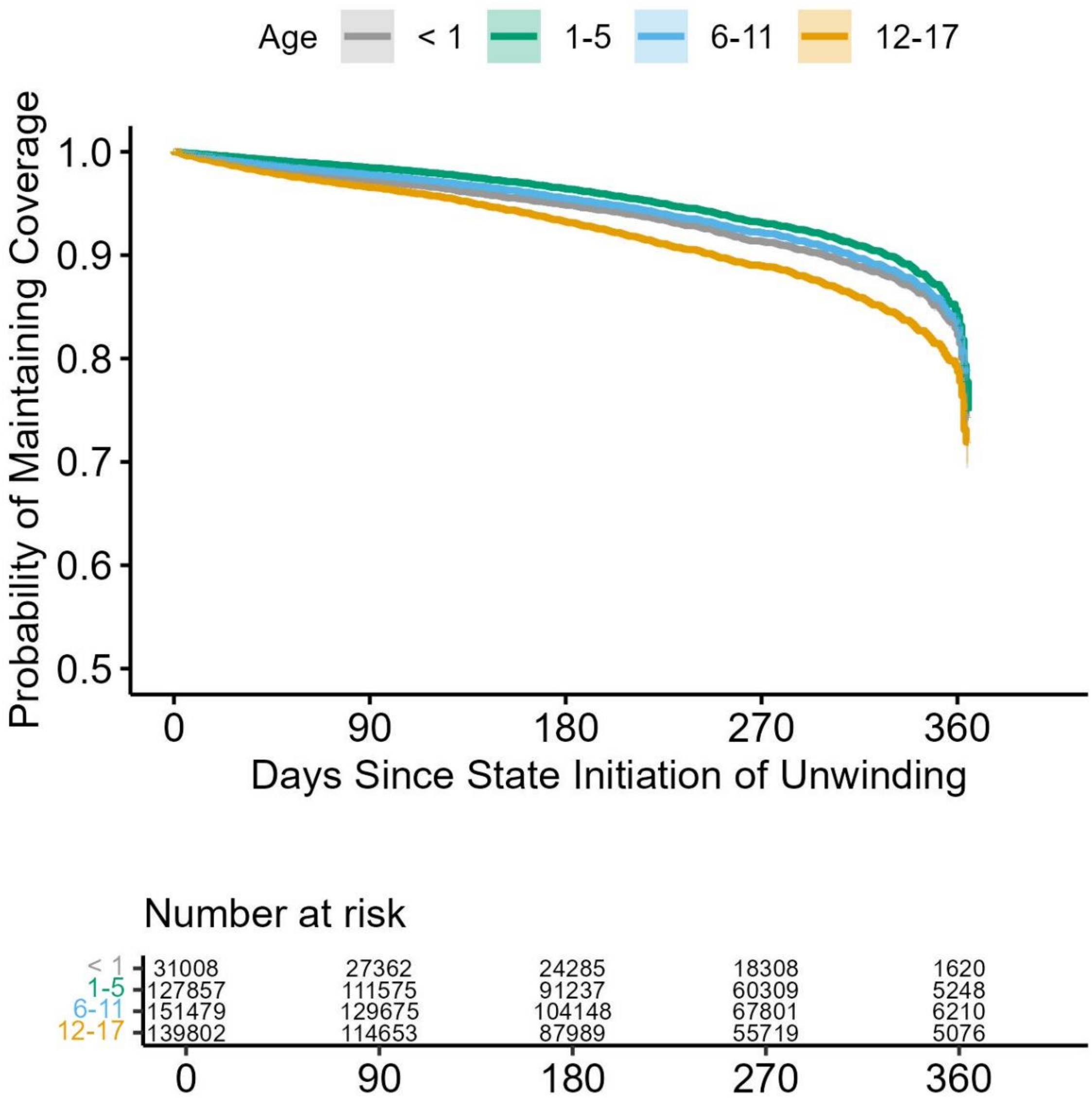

© 2025 Bensken WP et al. *JAMA Network Open*.

eFigure 4. Kaplan-Meier curve showing the unadjusted time-to-loss of coverage among Medicaid-insured pediatric patients, stratified by sex.

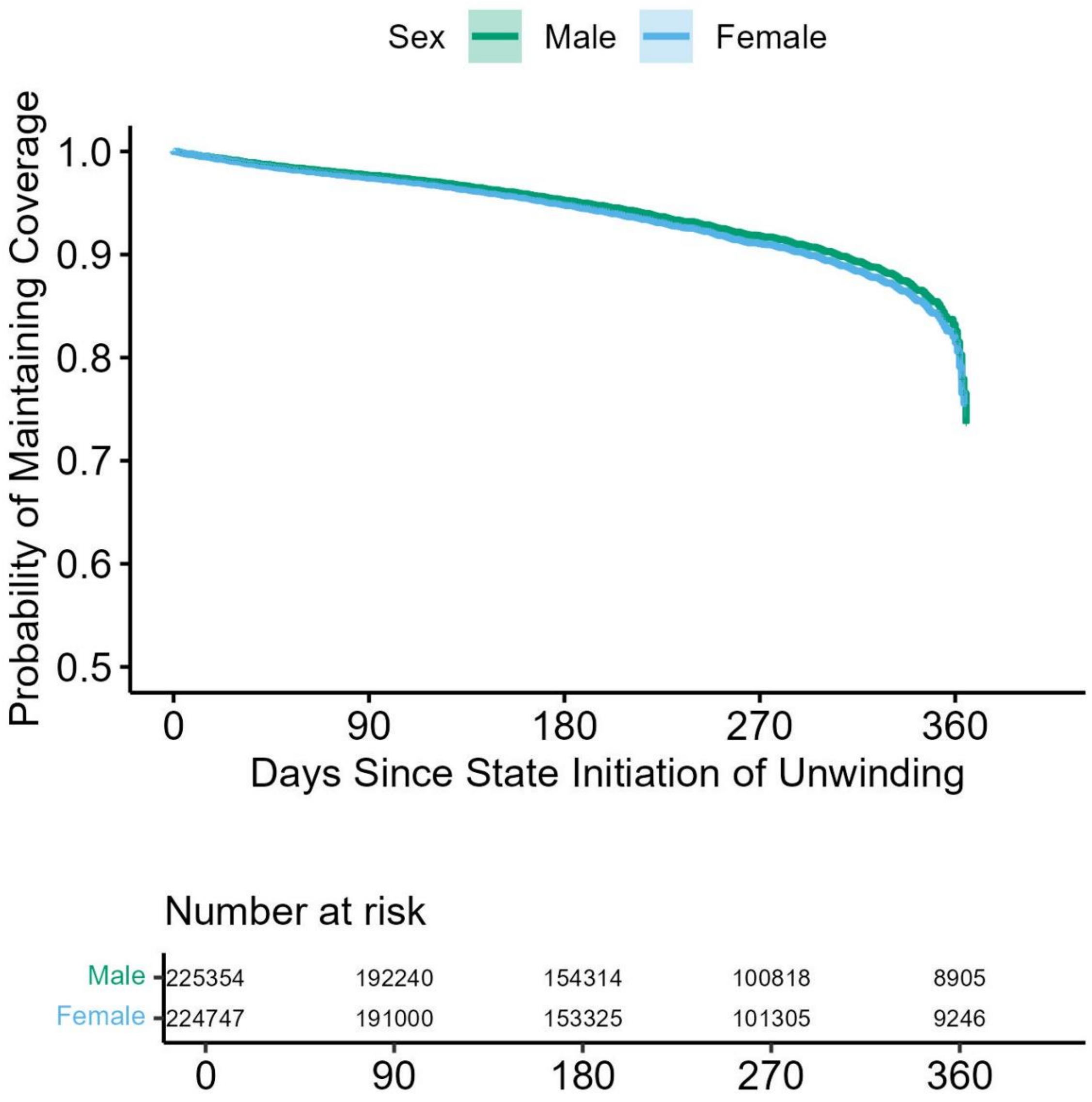

© 2025 Bensken WP et al. *JAMA Network Open*.

eFigure 5. Kaplan-Meier curve showing the unadjusted time-to-loss of coverage among Medicaid-insured pediatric patients, stratified by race/ethnicity.

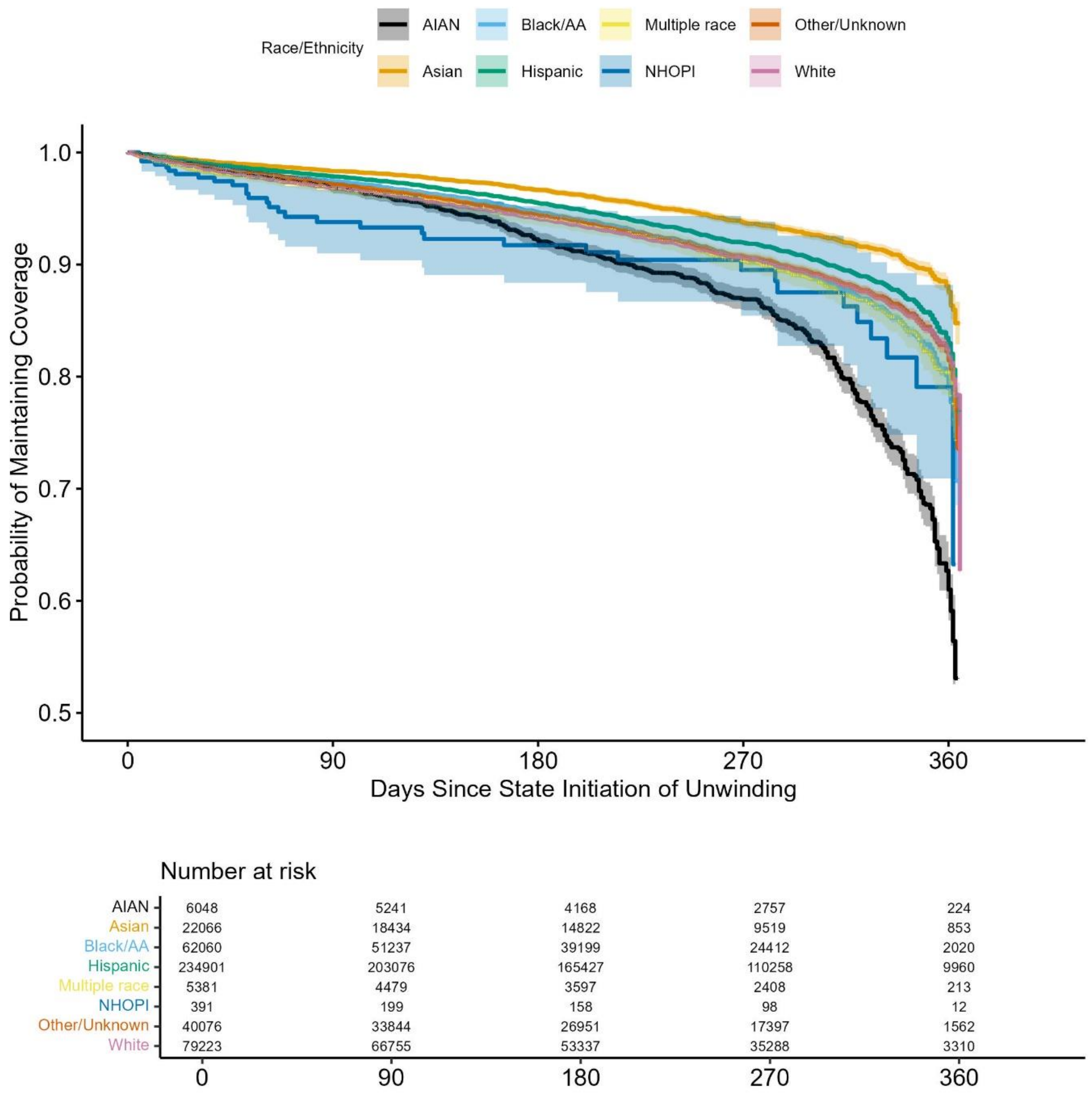

© 2025 Bensken WP et al. *JAMA Network Open*.

eFigure 6. Kaplan-Meier curve showing the unadjusted time-to-loss of coverage among Medicaid-insured pediatric patients, stratified by language

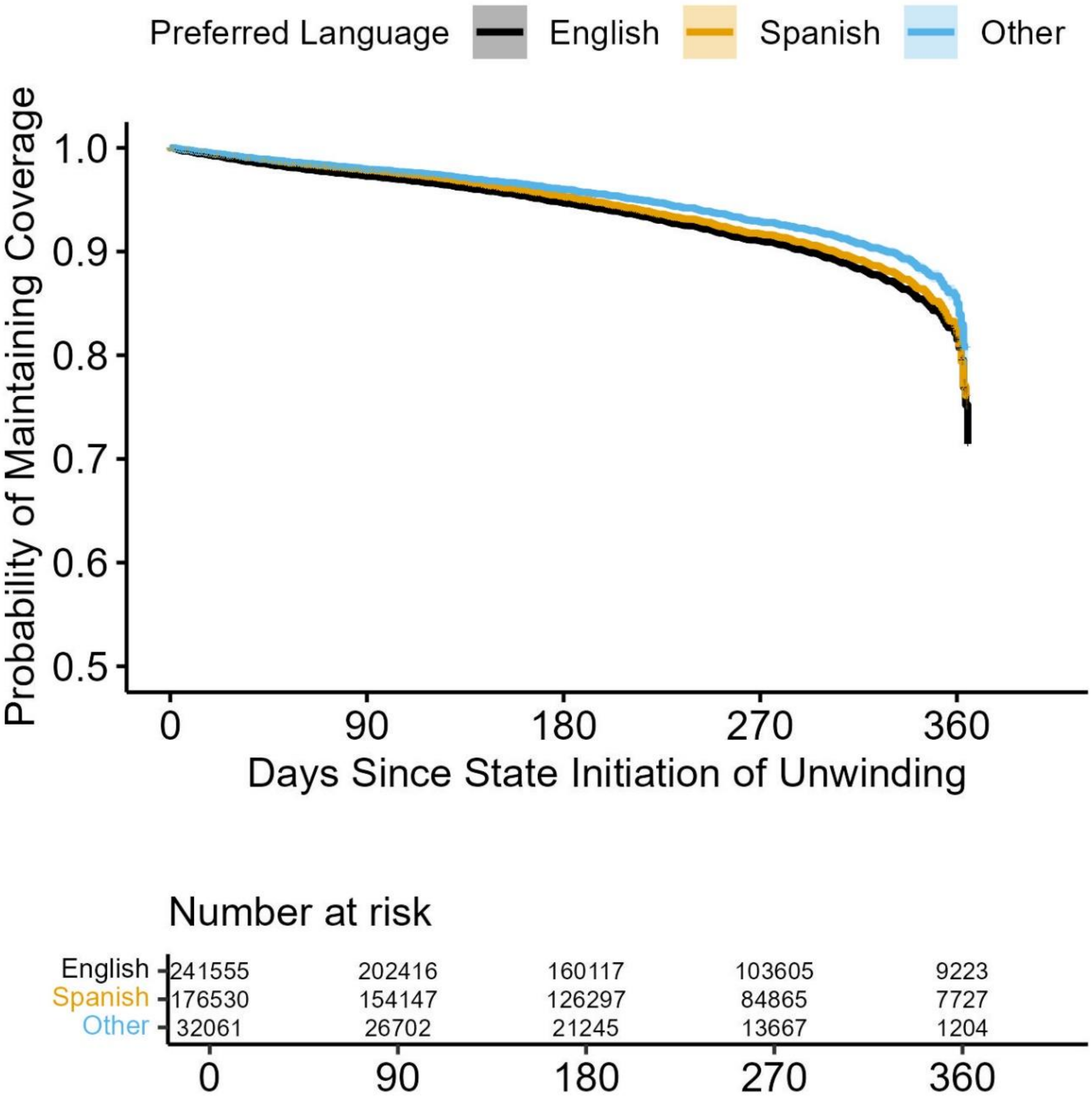

© 2025 Bensken WP et al. *JAMA Network Open*.

eFigure 7. Kaplan-Meier curve showing the unadjusted time-to-loss of coverage among Medicaid-insured pediatric patients, stratified by medical complexity

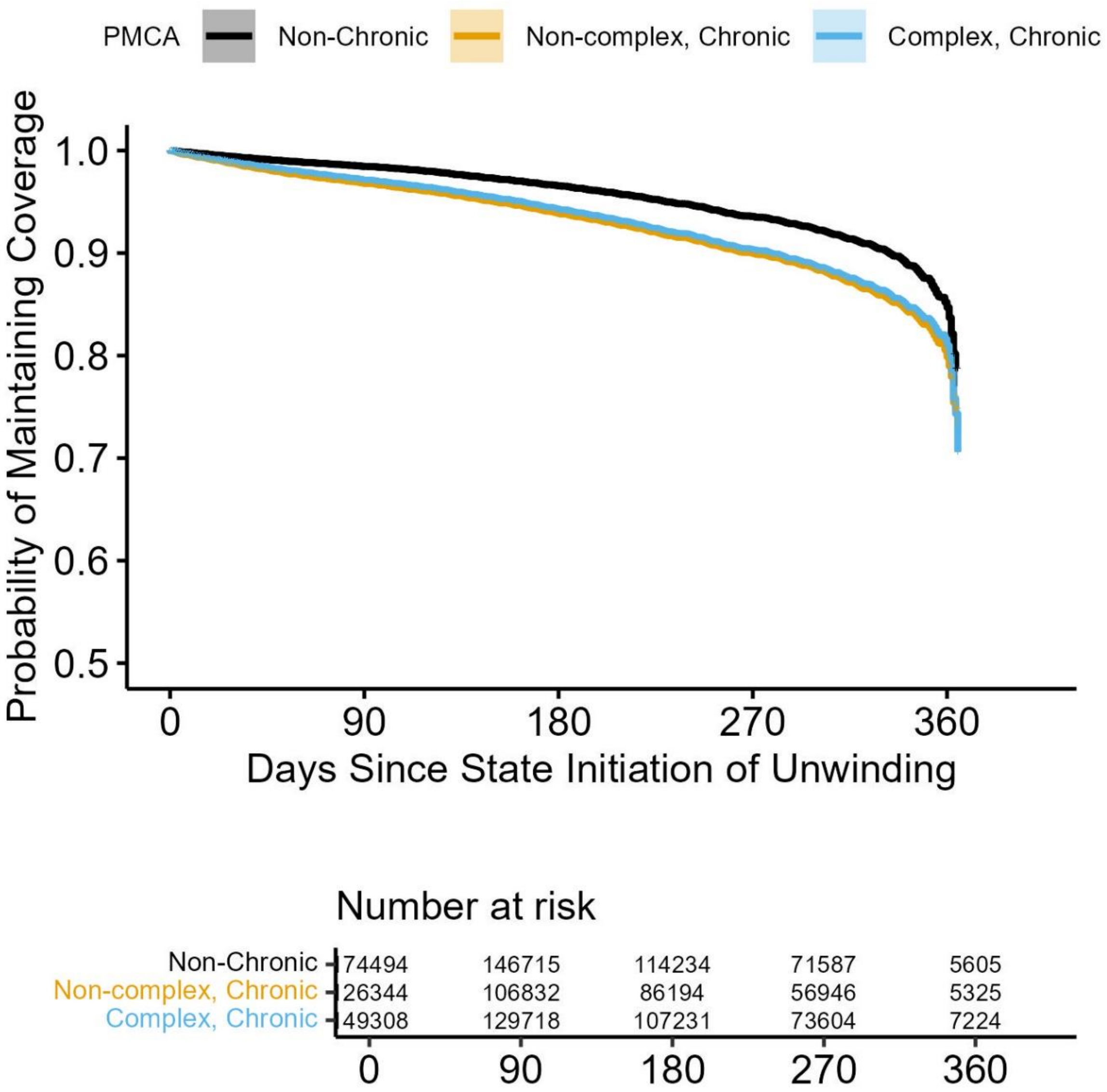

© 2025 Bensken WP et al. *JAMA Network Open*.
