# Supplementary material for: Unwinding of Continuous Medicaid Coverage Among Pediatric Community Health Center Patients
Source: JAMA Netw Open. 2025 Feb 6;8(2):e2458155. doi: 10.1001/jamanetworkopen.2024.58155 (PMC11803474; doi:10.1001/jamanetworkopen.2024.58155)
Supplement: Supplement 2. — Data Sharing Statement [file jamanetwopen-e2458155-s002.pdf]

Data Sharing Statement

Bensken. Unwinding of Continuous Medicaid Coverage Among Pediatric Community Health Center Patients. *JAMA Netw Open*. Published February 06, 2025.
doi:10.1001/jamanetworkopen.2024.58155

Data
Data available: No
